# Promoting Reflection on the Process of Recovery: Unique Contributions from Literature and the Humanities for Practitioner

**DOI:** 10.1007/s10597-024-01254-x

**Published:** 2024-03-05

**Authors:** Paul H. Lysaker, David Roe, John T. Lysaker

**Affiliations:** 1 Department of Psychiatry, Richard L. Roudebush VA Medical Center, Indianapolis, IN USA; 2 Department of Psychiatry, Indiana University School of Medicine, Indianapolis, IN USA; 3 Department of Community Mental Health, University of Haifa, Haifa, Israel; 4 Department of Philosophy, Emory University, Atlanta, GA USA

**Keywords:** Recovery, Serious mental illness, Humanities, Negative capability, Literature

## Abstract

Recovery from serious mental illness requires persons to make their own meaning and deal with evolving challenges and possibilities. Psychiatric rehabilitation thus must offer more than manualized curricula that address symptoms and skills. We suggest that exposure to the humanities and in particular literature may offer practitioners unique avenues for developing interventions that are sensitive to the processes that enable meaning to be made. We suggest that through what the poet Keats called negative capability, reading novels may enhance practitioners? abilities to see and accept uncertainty, tolerate ambiguity without need for complete resolution, and accept the complex and ambiguous nature of persons. As an illustration we described how reading two novels, The Trial and Slaughterhouse-Five enhanced the process of meaning making while supporting the recovery of one prototypical person with serious mental illness during his efforts to make sense of his experience of returning to work.

## Introduction

Recovery has played a major role in challenging older models of the course of serious mental illness (SMI). Research has shown that contrary to what was originally thought, many diagnosed with a SMI do not experience decline over time but rather recover in ways that vary from person to person (Leonhardt et al., 2017). Symptoms or signs of disorder may remit, and even when they do not or do so only partially, people are often able to find ways to cope and live a personally meaningful life.

Psychiatric rehabilitation has also spurred the development of a range of concrete interventions that promote recovery. Group, family, and individual interventions, for example, have been tested and found to effectively increase the likelihood of recovery by providing tailored supports that help develop needed skills and relationships which expand opportunities for community participation and empower the pursuit of goals and life visions (e.g., Kinoshita et al., 2010; McGuire et al., 2016; Mueser & Roe, 2018).

As research in psychiatric rehabilitation has stimulated the development of diverse interventions, it has emphasized that recovery is personal and involves self- direction. The recovery process requires that people develop an evolving sense of the challenges and possibilities they face and the capacity to choose the path(s) they wish to take. They must form a sense of what they want, what is possible, and what they want to do about it. This often involves the development of an evolving personal account or narrative of what has gone well and gone wrong, a feel for their own resilience, a sense of their desires, a grasp of their support networks, and an ability to imagine and try/develop/adapt novel coping strategies (Lysaker et al., 2020).

This growing awareness of the process of recovery has had far reaching implications for psychiatric rehabilitation. First, it has revealed that rehabilitation involves more than simply providing opportunities and teaching skills. No menu of services no matter how carefully defined can make up or compensate for a lack of self-direction. In addition, there is a need for psychiatric rehabilitation practitioners to engage in an active process in which the recovering person’s goals are formed, questioned, and revised in response to the recovering persons’ own interpretations and experiences across the process of rehabilitation (Hasson-Ohayon et al., 2017b; Roe et al., 2004).

This often requires practitioners to develop the ability to reflect upon how both their own thoughts and reactions and those of the recovering person may change in surprising ways and how the process of rehabilitation should evolve given those unexpected developments. Practitioners may need to remain fully open to themselves and those they work with, to discover unexpected things about themselves, the clinical setting, and the needs of their clients.

Such activities are complex. Because meaning and one’s place in the world may be at stake, no protocol, no matter how carefully developed, can anticipate in advance what meanings someone will make of what emerges in their lives as they recover (Roe & Lysaker, 2012). As noted by many including Kelly (1964), May (1958), and others, responding to life and discovering who we are as persons involves surprise and some degree of risk, including threat. Even success in making positive and desired changes is usually accompanied by pain, which can be hard but also generate new discoveries and a sense of meaning. Recovering persons, for example, may lose previously held identities, narratives, and values that guided past deliberations and choices (Buck et al., 2013). The process of self-direction itself may feel daunting as they become confused and/or uncertain about who they are and how they fit into the world (Lysaker et al., 2021). Moreover, their own accounts of what has happened to them in the past may diverge significantly from what practitioners have assumed to be true, thus intensifying confusion (Roe et al., 2021; Rotstein et al., 2018; Rosenthal-Oren et al., 2021).

As an illustration, working part-time and finding a job in the community might seem like a simple goal. For some, working might mean greater financial stability or improved self-esteem, as expected. These experiences may, however, also accrue meanings that are unexpected. Working also may mean that one is overcoming past challenges, raising the specter of what those challenges were, what they suggest about one’s character, and what overcoming them means.

Working may further change the meaning of past achievements, losses, injustices, and future dreams, rendering the meanings the recovery process can have as something that evolves fluidly, provoking any number of powerful emotional reactions (Roe, 2017).

We think that much of the science which forms the backbone of psychiatric rehabilitation may have diminishing returns in these moments of transition and transformation. The nomothetic approaches which guide psychiatric rehabilitation may provide important information about how likely it is that a particular intervention may lead to a certain outcome, but less insight into what the activities and processes of recovery are coming to mean to mean to person (Lysaker & Roe, 2013). Worse, it risks usurping the process of meaning making by providing a predetermined, often over-generalized framework that assumes what people believe, desire and should think about their dilemmas and how to resolve them (Hasson-Ohayon et al., 2017a).

We believe that broad exposure to the humanities may help clinicians remain open and nimble as clients work through their experiences in recovery (Roe & Lysaker, in press a). The humanities immerse us in the evolving process of meaning making and surprise, and they open, without relying on formulaic solutions, powerful windows into concrete human dilemmas regarding suffering, growth/change, and self-discovery within one’s own community. In a recent paper (Roe & Lysaker, in press b) we argued that jazz, a form of art which entails both structure and improvising, may enrich and broaden clinicians’ abilities to facilitate meaning making with clients to promote recovery. *W*e explored how jazz may be a space where specific processes can be observed and accordingly guide psychotherapy focused on subjective forms of recovery. We discovered that jazz offers a space to see how timing, risk-taking, the ability to be simultaneously inside and outside an activity, and support for the process of tension and release can inform and inspire the process of improvisation within psychotherapy.

While this is true about many projects in the humanities, in this paper we will focus on reading novels, and on Keats’ notion of negative capability. Specifically, we will explore how novels may enhance psychosocial rehabilitation practitioners’ abilities to help recovering people develop an evolving sense of the challenges and paths they face by allowing practitioners to openly accept and respond to the uncertainty which naturally emerges within the recovery process.

To consider this possibility, we will first explore the concept of negative capability and how it may foster three related kinds of abilities among practitioners: the ability to accept uncertainty, the ability to respond to uncertainty in ways which allow for meaning to emerge, and the ability to understand the complexities which are inherent in anyone’s emerging responses to life. To illustrate this possibility, we will describe how reading two particular novels concretely offered opportunities for making sense of the evolving experience of rehabilitation and recovery. Finally, we will contrast this view with the use of literature as a case example or a mock first-person account and highlight the former’s potentially distinct contributions. Of note, a broad literature exists concerning bibliotherapy, or the effects of exposure among recovering persons to differing kinds of literature (e.g. Gregory et al., 2004). Our explicit purpose however is to focus on its distinctive effects upon practitioners and not as a treatment component meant to support recovering persons.

## Keats and Negative Capability

Writing to his brothers in 1817, the English poet John Keats (1899) praised Shakespeare for his ability to acknowledge nuance, conflict, and uncertainty while exploring the complexities of the world in drama and poetry. He termed this ability: “Negative Capability, that is when man is capable of being in uncertainties, mysteries, doubts, without any irritable reaching after fact and reason.” (Keats, 1899). The point is not to simply maintain uncertainty, however, but to act in the face of it, to venture meanings while “remaining content with half-knowledge.” (Keats, 1899) While Keats has writing in mind, his observation applies to reading and, more generally, finding and making meaning out of life experience. Negative capability is the ability to experience uncertainty about the world we inhabit and the possibilities it affords and to respond imaginatively.

Negative capability has been explored in a range of different contexts, including the reading and writing of literature. Our suggestion is that those who exercise negative capability when reading literature might strengthen their ability to participate with recovering persons in the joint development of an evolving sense of the challenges and possibilities they face and the path(s) they wish to take or avoid.

One capacity strengthened through the exercise of negative capability in awareness of a patience with experiences of ambiguity, doubt, and vagueness. With negative capability we can respond to uncertainty in a nonjudgmental manner and even assume its inevitability. In literature the reader always encounters a period of not knowing and the reader is required to suspend any nagging need for initial clarity or certainty. Reading the sentences of a novel, we have some sense of the characters, but we do not know how they got where they are, what moves them, and where they are headed. So too with the recovering person during rehabilitation. We must recognize and be patient with our not fully knowing what meanings will emerge for them.

An ability to patiently endure uncertainty, one potentially cultivated by reading novels, may assist the practitioner to accept that the value of any given outcome may be uncertain. It cannot be determined or known ahead of time based on a predetermined protocol. With negative capability, practitioners can accept that the meaning of recovery can always emerge unpredictably and may not be fully knowable in any given moment. Keats’ use of the phrase “irritable reaching after fact and reason” and “being content with half-knowledge” suggests that with negative capability, practitioners should not find this uncertainty averse but instead be open to the possibilities that might emerge from that uncertainty within ongoing dialogue with recovering persons about their experiences. As a result, negative capacity should encourage a kind of caution and even hesitancy when tempted to prematurely resolve uncertainties. One should be averse to simplistic formulations when trying to jointly make sense of the suffering and possibilities experienced by the recovering person. This is consistent with observations that supervision groups which include the reading and discussion of literature (Leonhardt et al., 2015) and film (Roe, 2020) may help mental health professionals master their own negative reactions to ambiguity in general). This is also consistent with psychodynamic reflection about the need to continuously suspend certainty and the conclusions one has previously drawn about persons in psychotherapy (e.g., Bion, 1970).

While negative capability may breed the ability to acknowledge uncertainty, it also may allow for action and meaning making in the face of that uncertainty. As we read novels, we do not passively await the resolution of uncertainty but rather actively think about and react as the actions unfold, affirming or frustrating our sense of what is happening in the work; so it is in the search for meaning within the recovery process. Not only does the clinician accept uncertainty but they seek meaning without knowing ahead of time whether they are on the right track. They take risks as they trust a hunch and wonder whether something is happening and follow up and see how the recovering persons reacts, a process that must be repeated continuously as mutual understanding emerges between the recovering person and the rehabilitation specialist. Negative capability thus allows for a process of discovery and invention which may lead to new experiences that require new discoveries and inventions in future sessions.

In the construction of meaning, Keats suggests that, at least with great poets, the “sense of Beauty overcomes every other consideration, or rather, obliterates all consideration.” (Keats, 1899) While “Beauty” is not the goal of the recovery process, Keats’s observation is still instructive. He has in mind a view of the whole as greater than the sum of its parts and suggests that keeping that whole in view allows for a richer construction of meaning. In our view, this does apply to rehabilitation therapies, where clinicians must see beyond details and help clients find identities and possibilities in complex, shifting experiences. Appreciating holistic conceptions of meaning making also positions meaning making as not merely the top of a hierarchy of needs but more in line with Frankl (1965) as something that makes it possible to endure significant degrees of suffering and make considerable sacrifices for what one truly values. We would suggest that this aspect of negative capability would allow a rehabilitation specialist to continually recontextualize an individual’s achievements and setbacks within their larger, evolving sense of self and life projects. This is consistent with observations about intersubjective processes allows for joint meaning within the process of recovery from serious mental illness (Hamm et al., 2021; Hasson-Ohayon et al., 2020).

A third, related point is that with negative capability there may come an enhanced ability to not merely be patient and holistic while jointly constructing meaning but also to see the recovering person and themselves as full of inherent contradictions and complexities as they jointly seek meaning in the course of recovery. As the poet Simic (1985) notes, literature promotes the ability to see ourselves as both a “kind of magnet for complex, historical, literacy and psychological forces, as well as a way of maintaining oneself in the face of that multiplicity” (p. 83). This echoes the observations of Bakhtin (1929) that literature opens a path to understanding how any human life is always a matter of dialogue, both within and between persons which includes contradictory, complementary, and unrelated aspects. Thus, with negative capacity it would seem that rehabilitation specialists could understand the complex forces which influence and often shape the unique experience of a recovering person. This would enable joint reflection about the unique meaning that various accomplishments or setbacks could have within the recovery process. It is also consistent with reflections that one key aspect of the psychosocial treatments for persons recovering from SMI is the need not to collude with fragmentation or accept partial singular aspects of experience as fully defining the recovering person (Hamm et al., 2017).

## Literature, Negative Capability, and Recovery

To explore the points above more deeply we turn to how two novels, *The Trial* by Franz Kafka and *Slaughterhouse-Five* by Kurt Vonnegut, read through an exercise in negative capability, may have strengthened the first author’s ability (PHL) to work with recovering persons as they develop an evolving sense of self and act upon the challenges and paths they faced in life. These are two of the novels read by PHL in quarterly meetings in which professionals and their students involved in recovery read and discussed how one book per meeting affected how they thought about and delivered psychosocial rehabilitation services. They are not intended to represent the most important novels to read but to serve as examples of the process. *The Trial*, written in 1914 and 1915 details the harrowing experiences of the main character who attempts to engage the justice system after he is accused of and tried for a crime which is never named. *Slaughterhouse-Five*, written in 1969, follows a main character who, having survived the bombing of Dresden as a captured American solider, returns from World War II and encounters a bizarre, extraterrestrial race.

As noted above, the first aspect of negative capability relevant to recovery practice is the ability to know and accept uncertainty. Here we suggest that both novels helped PHL to get a feeling for the uncertainties that may emerge during recovery and to patiently allow those uncertainties to unfold, resisting any anxious urge to resolve them before their meaning could be known. In *The Trial*, negative capability is exercised as the reader cannot know what crime the character in *The Trial* supposedly committed, whether it ever happened, and how the character will work through their presumed guilt. In *Slaughterhouse-Five*, negative capability is required as the reader thinks beyond the particular descriptions to imagine the massive toll on civilian life brought about by the firebombing of Dresden, aware that there was no apparent reason for why the onslaught was necessary.

Turning the context of recovery, we’d like to consider the case of one adult male we will call Gordon. Gordon wanted to return to work after being on disability for over a decade. He expected to earn money and feel more involved in his community. What emerged after a brief period was that social interactions were complicated and confusing for him and seemed to point to the possibilities that others would never accept him, that all past vocational struggles were solely his fault, and that others mostly wanted him to fail and should be confronted. At the same time, working also raised many questions about Gordon’s life which had no clear answers. Did success at work mean Gordon had wasted the years of his life during which he had not worked? Allowing for uncertainty, as one must when reading *The Trial* and *Slaughterhouse-Five*, PHL was able to accept the uncertainty that Gordon faced as he struggled to make sense of surprising experiences that in turn generated complex and seemingly negative thoughts and feelings. Rather than moving quickly to supply answers, PHL could join Gordon in the uncertainty he faced, acknowledge its complexity and the pain it brought about, and patiently await whatever meaning emerged even in the absence of a solution to a concrete problem.

The second aspect of negative capability related to recovery is that persons are able to venture responses to confusion uncertain about whether those responses are entirely correct. Concerning *The Trial* and *Slaughterhouse-Five*, neither novel has what could be considered a happy ending that resolves all confusion and injustice. And any reading of either novel concludes with its own unresolved sense of what exactly happened and why. Nevertheless, both allow for the painful and overwhelming dilemmas faced by the protagonists to be named and responded to by the reader. In our illustration, as uncertainty about work emerged in positive and difficult experiences, Gordon found himself wondering if working would mean that the traumatic experience of psychosis and social exclusion was over, and to the point that nothing more needed to be said about it. But that of course could not be determined ahead of time, and so Gordon had to make decisions about whether to keep working from a position of what Keats terms half-knowledge. Through reading *The Trial* and *Slaughterhouse-Five*, PHL also had to continually engage Gordon from a position of uncertainty about what Gordon was likely to experience regardless of what he elected to do. Our suggestion is not that the novels gave PHL insight into Gordon’s particular experiences. Rather, reading them strengthened PHL’s ability to work with Gordon amid inevitable uncertainties and the risks they posed, both for Gordon and for the recovery process. Importantly, the meaning making that took place in our illustration did not occur in a single transaction. It occurred incrementally and relative to larger conceptions of who Gordon was, who he wanted to be, and always in terms of what one can expect in a complex world. And, as with reading both novels, there was no assumption that there would be one answer that resolved Gordon’s suffering. Questions always persist and so it was the process of meaning making itself that made living with that suffering meaningful and understandable as a natural part of the human condition.

The final aspect of negative capability that reading novels involves an increasing recognition of and appreciation for the complexity and uncertain character of who and what we are as persons. In reading novels we accept contradictions within the characters that cannot be singularly resolved. In both *The Trial and Slaughterhouse-Five* the characters have multiple facets which can be contradictory, complementary, or unrelated. The characters have, for example, erotic, aggressive, compassionate, egocentric, and moral aspects of themselves, none of which is primary. Similarly in our illustration, Gordon could be said to have the same collection of facets which the rehabilitation specialist could understand as co-existing. And just as appreciating that complexity is a part of coming to know the characters in novel, so too, without any need to explain them with a single, unifying variable, PHL realized that something similar was required if he was to know Gordon. And it was this kind of complex half-knowledge that enabled even deeper conversations about the meanings that returning to work had for Gordon including sexual desires, a wish for a prominent social position, and a need to care for others.

## Discussion and Conclusions

In this paper we have suggested that as the vision of recovery has evolved, it has posed a set of difficulties which exceed the direction of manualized interventions. These include persons making their own unique meaning of recovery, including its challenges and successes. Here we have suggested that the humanities may offer something unique. Through what Keats called negative capability we have argued that reading novels may enhance practitioners’ abilities to see and accept uncertainty, act within that uncertainty without needing complete resolution or certainty, and accept the uncertain nature of persons themselves. As an illustration we described how reading two novels, *The Trial* and *Slaughterhouse-Five*, enhanced that process of meaning which occurred as one prototypical person with SMI returned to work.

One could ask though whether these reflections offer anything unique since literature has long been suggested as offering value in the allied fields of mental health through case examples or fictionalized accounts of first-person experience (Silberger, 1973; Tucker, 1994). We would suggest that this view does depart from these older models in several ways. Foremost amongst these is the idea that novels help us see how to make meaning rather than simply deferring to a set of predetermined meanings. Reading novels helps us to know and move within uncertainty and to focus on the process of meaning making itself. In this way they help practitioners listen to themselves as they listen to recovering persons, magnifying and possibly strengthening the kinds of activities that allow for meaning to made within recovery without rejecting some of the key structures of rehabilitation itself.

Importantly, there are limitations to our inquiry, which also raises new questions. We have focused on novels and certainly much could be said about other arts and humanities including music, film, dance, fine arts, poetry etc. We also have not discussed the various arenas or forums in which practitioners support the recovery process. There remains much to explore concerning what exposure to the arts with other practitioners, solo exposure, or exposure with other groups including recovering persons adds to this process. The arts are also a culturally embedded phenomenon and what one art means to one practitioner might differ from what it means to another. The novels described here, for example, would likely mean different things to practitioners with differing cultures, backgrounds etc. More careful work is thus needed to think about the interaction of the humanities and art with unique practitioners. For example, what makes one particular novel or art form more helpful than another given the personal characteristics and background of the clinician?
